# Supplementary material for: Heparin-based hydrogel scaffolding alters the transcriptomic profile and increases the chemoresistance of MDA-MB-231 triple-negative breast cancer cells
Source: Biomater Sci. 2020 Feb 13;8(10):2786–96. doi: 10.1039/c9bm01481k (PMC7497406; doi:10.1039/c9bm01481k)
Supplement: Supplementary file 2 [file BM-008-C9BM01481K-s002.zip › Supplementary File 4/HP-B hydrogel + EGF (s)vControl/Pathways/my_analysis.Gsea.1545201170987/HALLMARK_INTERFERON_GAMMA_RESPONSE.html]

Details for gene set HALLMARK\_INTERFERON\_GAMMA\_RESPONSE[GSEA]

|  || Dataset | expr.class.cls#HYDEGF\_versus\_CONTROL.class.cls#HYDEGF\_versus\_CONTROL\_repos |
| Phenotype | class.cls#HYDEGF\_versus\_CONTROL\_repos |
| Upregulated in class | CONTROL |
| GeneSet | HALLMARK\_INTERFERON\_GAMMA\_RESPONSE |
| Enrichment Score (ES) | -0.50835246 |
| Normalized Enrichment Score (NES) | -2.3818688 |
| Nominal p-value | 0.0 |
| FDR q-value | 0.0 |
| FWER p-Value | 0.0 |
Table: GSEA Results Summary

  

Fig 1: Enrichment plot: HALLMARK\_INTERFERON\_GAMMA\_RESPONSE      
 Profile of the Running ES Score & Positions of GeneSet Members on the Rank Ordered List

  

| PROBE | DESCRIPTION (from dataset) | GENE SYMBOL | GENE\_TITLE | RANK IN GENE LIST | RANK METRIC SCORE | RUNNING ES | CORE ENRICHMENT || 1 | IL18BP | na |  |  | 434 | 2.192 | -0.0085 | No |
| 2 | UPP1 | na |  |  | 814 | 1.811 | -0.0165 | No |
| 3 | TRIM14 | na |  |  | 1248 | 1.540 | -0.0292 | No |
| 4 | PARP12 | na |  |  | 1533 | 1.416 | -0.0348 | No |
| 5 | LATS2 | na |  |  | 1663 | 1.369 | -0.0326 | No |
| 6 | NOD1 | na |  |  | 1782 | 1.335 | -0.0301 | No |
| 7 | RNF213 | na |  |  | 2359 | 1.162 | -0.0528 | No |
| 8 | ZNFX1 | na |  |  | 2629 | 1.096 | -0.0597 | No |
| 9 | HERC6 | na |  |  | 2887 | 1.030 | -0.0665 | No |
| 10 | IRF7 | na |  |  | 2931 | 1.020 | -0.0621 | No |
| 11 | OASL | na |  |  | 3235 | 0.954 | -0.0717 | No |
| 12 | RBCK1 | na |  |  | 3630 | 0.880 | -0.0867 | No |
| 13 | DHX58 | na |  |  | 3730 | 0.859 | -0.0863 | No |
| 14 | MARCH1 | na |  |  | 3831 | 0.841 | -0.0860 | No |
| 15 | NLRC5 | na |  |  | 3944 | 0.822 | -0.0865 | No |
| 16 | SLC25A28 | na |  |  | 4291 | 0.768 | -0.0997 | No |
| 17 | ISG15 | na |  |  | 4664 | 0.711 | -0.1146 | No |
| 18 | JAK2 | na |  |  | 4877 | 0.671 | -0.1213 | No |
| 19 | RAPGEF6 | na |  |  | 4984 | 0.654 | -0.1226 | No |
| 20 | TRIM26 | na |  |  | 4988 | 0.653 | -0.1185 | No |
| 21 | MX1 | na |  |  | 5059 | 0.644 | -0.1179 | No |
| 22 | CMPK2 | na |  |  | 5304 | 0.604 | -0.1268 | No |
| 23 | TRIM25 | na |  |  | 5383 | 0.593 | -0.1270 | No |
| 24 | USP18 | na |  |  | 5430 | 0.586 | -0.1256 | No |
| 25 | IFI30 | na |  |  | 5554 | 0.570 | -0.1283 | No |
| 26 | PTPN6 | na |  |  | 5592 | 0.564 | -0.1266 | No |
| 27 | CD274 | na |  |  | 5686 | 0.548 | -0.1279 | No |
| 28 | RSAD2 | na |  |  | 5911 | 0.515 | -0.1363 | No |
| 29 | STAT2 | na |  |  | 6213 | 0.474 | -0.1490 | No |
| 30 | FGL2 | na |  |  | 6216 | 0.474 | -0.1460 | No |
| 31 | LYSMD2 | na |  |  | 6312 | 0.461 | -0.1480 | No |
| 32 | RNF31 | na |  |  | 6324 | 0.460 | -0.1455 | No |
| 33 | PELI1 | na |  |  | 6361 | 0.456 | -0.1444 | No |
| 34 | SLAMF7 | na |  |  | 6581 | 0.427 | -0.1531 | No |
| 35 | IFI44L | na |  |  | 6789 | 0.397 | -0.1614 | No |
| 36 | PNP | na |  |  | 6968 | 0.376 | -0.1683 | No |
| 37 | OGFR | na |  |  | 7200 | 0.343 | -0.1782 | No |
| 38 | IL7 | na |  |  | 7239 | 0.338 | -0.1780 | No |
| 39 | OAS3 | na |  |  | 7492 | 0.305 | -0.1892 | No |
| 40 | CIITA | na |  |  | 7521 | 0.302 | -0.1887 | No |
| 41 | NUP93 | na |  |  | 7792 | 0.264 | -0.2012 | No |
| 42 | DDX60 | na |  |  | 7899 | 0.256 | -0.2051 | No |
| 43 | PML | na |  |  | 7928 | 0.252 | -0.2049 | No |
| 44 | PTPN2 | na |  |  | 8085 | 0.230 | -0.2116 | No |
| 45 | IFNAR2 | na |  |  | 8233 | 0.217 | -0.2179 | No |
| 46 | NFKB1 | na |  |  | 8333 | 0.205 | -0.2217 | No |
| 47 | ADAR | na |  |  | 8616 | 0.170 | -0.2354 | No |
| 48 | MTHFD2 | na |  |  | 8784 | 0.152 | -0.2432 | No |
| 49 | ITGB7 | na |  |  | 8884 | 0.137 | -0.2475 | No |
| 50 | IFI35 | na |  |  | 9363 | 0.080 | -0.2721 | No |
| 51 | PTPN1 | na |  |  | 9696 | 0.050 | -0.2892 | No |
| 52 | SAMD9L | na |  |  | 9711 | 0.048 | -0.2896 | No |
| 53 | IFIT2 | na |  |  | 9862 | 0.027 | -0.2973 | No |
| 54 | IFI44 | na |  |  | 9863 | 0.027 | -0.2972 | No |
| 55 | XAF1 | na |  |  | 10004 | 0.020 | -0.3044 | No |
| 56 | IFIH1 | na |  |  | 10011 | 0.019 | -0.3046 | No |
| 57 | SECTM1 | na |  |  | 10155 | -0.003 | -0.3121 | No |
| 58 | IL15RA | na |  |  | 10423 | -0.038 | -0.3259 | No |
| 59 | PFKP | na |  |  | 10539 | -0.041 | -0.3316 | No |
| 60 | CASP8 | na |  |  | 11048 | -0.114 | -0.3576 | No |
| 61 | IRF1 | na |  |  | 11060 | -0.115 | -0.3574 | No |
| 62 | PIM1 | na |  |  | 11181 | -0.119 | -0.3629 | No |
| 63 | PNPT1 | na |  |  | 11210 | -0.124 | -0.3636 | No |
| 64 | SAMHD1 | na |  |  | 11247 | -0.130 | -0.3646 | No |
| 65 | RIPK2 | na |  |  | 11399 | -0.146 | -0.3716 | No |
| 66 | STAT1 | na |  |  | 11412 | -0.148 | -0.3713 | No |
| 67 | BANK1 | na |  |  | 11768 | -0.198 | -0.3886 | No |
| 68 | SP110 | na |  |  | 11857 | -0.213 | -0.3919 | No |
| 69 | SSPN | na |  |  | 12019 | -0.219 | -0.3989 | No |
| 70 | IFIT1 | na |  |  | 12079 | -0.229 | -0.4005 | No |
| 71 | CFH | na |  |  | 12170 | -0.240 | -0.4036 | No |
| 72 | IL4R | na |  |  | 12312 | -0.263 | -0.4093 | No |
| 73 | CASP1 | na |  |  | 12497 | -0.286 | -0.4171 | No |
| 74 | SOCS3 | na |  |  | 12654 | -0.306 | -0.4233 | No |
| 75 | MYD88 | na |  |  | 12655 | -0.306 | -0.4213 | No |
| 76 | IL15 | na |  |  | 12671 | -0.309 | -0.4201 | No |
| 77 | MVP | na |  |  | 13105 | -0.354 | -0.4405 | No |
| 78 | PSMB10 | na |  |  | 13119 | -0.356 | -0.4389 | No |
| 79 | ARL4A | na |  |  | 13134 | -0.358 | -0.4373 | No |
| 80 | VCAM1 | na |  |  | 13271 | -0.382 | -0.4419 | No |
| 81 | CD40 | na |  |  | 13369 | -0.398 | -0.4444 | No |
| 82 | SPPL2A | na |  |  | 13751 | -0.449 | -0.4615 | No |
| 83 | FAS | na |  |  | 13787 | -0.456 | -0.4603 | No |
| 84 | IRF9 | na |  |  | 13794 | -0.458 | -0.4577 | No |
| 85 | PARP14 | na |  |  | 13951 | -0.479 | -0.4627 | No |
| 86 | IFIT3 | na |  |  | 13997 | -0.489 | -0.4619 | No |
| 87 | MX2 | na |  |  | 14127 | -0.494 | -0.4654 | No |
| 88 | PLSCR1 | na |  |  | 14338 | -0.526 | -0.4730 | No |
| 89 | FPR1 | na |  |  | 14461 | -0.549 | -0.4758 | No |
| 90 | CCL2 | na |  |  | 14503 | -0.557 | -0.4743 | No |
| 91 | RTP4 | na |  |  | 14707 | -0.591 | -0.4811 | No |
| 92 | HLA-DQA1 | na |  |  | 14720 | -0.591 | -0.4779 | No |
| 93 | GCH1 | na |  |  | 14797 | -0.603 | -0.4779 | No |
| 94 | TOR1B | na |  |  | 14817 | -0.607 | -0.4750 | No |
| 95 | TDRD7 | na |  |  | 14836 | -0.610 | -0.4719 | No |
| 96 | RIPK1 | na |  |  | 14875 | -0.615 | -0.4699 | No |
| 97 | CDKN1A | na |  |  | 14882 | -0.616 | -0.4662 | No |
| 98 | CXCL10 | na |  |  | 14952 | -0.628 | -0.4657 | No |
| 99 | PSMA3 | na |  |  | 15431 | -0.706 | -0.4862 | No |
| 100 | PSMA2 | na |  |  | 15449 | -0.711 | -0.4824 | No |
| 101 | CASP7 | na |  |  | 15674 | -0.757 | -0.4892 | No |
| 102 | EIF2AK2 | na |  |  | 16039 | -0.830 | -0.5029 | Yes |
| 103 | STAT3 | na |  |  | 16112 | -0.847 | -0.5012 | Yes |
| 104 | VAMP5 | na |  |  | 16233 | -0.881 | -0.5017 | Yes |
| 105 | NFKBIA | na |  |  | 16276 | -0.892 | -0.4981 | Yes |
| 106 | LY6E | na |  |  | 16278 | -0.893 | -0.4923 | Yes |
| 107 | TRIM21 | na |  |  | 16298 | -0.897 | -0.4874 | Yes |
| 108 | TAPBP | na |  |  | 16305 | -0.899 | -0.4818 | Yes |
| 109 | SRI | na |  |  | 16360 | -0.913 | -0.4787 | Yes |
| 110 | LGALS3BP | na |  |  | 16379 | -0.918 | -0.4736 | Yes |
| 111 | MT2A | na |  |  | 16590 | -0.967 | -0.4783 | Yes |
| 112 | PLA2G4A | na |  |  | 16752 | -1.011 | -0.4802 | Yes |
| 113 | PSMB2 | na |  |  | 16760 | -1.014 | -0.4739 | Yes |
| 114 | NCOA3 | na |  |  | 16858 | -1.044 | -0.4722 | Yes |
| 115 | CFB | na |  |  | 16882 | -1.049 | -0.4665 | Yes |
| 116 | IFITM3 | na |  |  | 16890 | -1.051 | -0.4600 | Yes |
| 117 | TNFAIP3 | na |  |  | 16896 | -1.053 | -0.4534 | Yes |
| 118 | TRAFD1 | na |  |  | 16979 | -1.082 | -0.4506 | Yes |
| 119 | TAP1 | na |  |  | 16995 | -1.089 | -0.4442 | Yes |
| 120 | HLA-G | na |  |  | 17031 | -1.100 | -0.4389 | Yes |
| 121 | EIF4E3 | na |  |  | 17034 | -1.100 | -0.4318 | Yes |
| 122 | APOL6 | na |  |  | 17142 | -1.126 | -0.4300 | Yes |
| 123 | ICAM1 | na |  |  | 17147 | -1.127 | -0.4229 | Yes |
| 124 | PDE4B | na |  |  | 17197 | -1.143 | -0.4180 | Yes |
| 125 | HLA-DMA | na |  |  | 17221 | -1.152 | -0.4116 | Yes |
| 126 | HIF1A | na |  |  | 17317 | -1.190 | -0.4088 | Yes |
| 127 | CASP3 | na |  |  | 17375 | -1.207 | -0.4039 | Yes |
| 128 | LAP3 | na |  |  | 17397 | -1.213 | -0.3971 | Yes |
| 129 | WARS | na |  |  | 17398 | -1.213 | -0.3891 | Yes |
| 130 | DDX58 | na |  |  | 17467 | -1.244 | -0.3846 | Yes |
| 131 | IFITM2 | na |  |  | 17505 | -1.262 | -0.3782 | Yes |
| 132 | UBE2L6 | na |  |  | 17509 | -1.262 | -0.3701 | Yes |
| 133 | HLA-B | na |  |  | 17516 | -1.266 | -0.3622 | Yes |
| 134 | C1R | na |  |  | 17523 | -1.268 | -0.3542 | Yes |
| 135 | CMKLR1 | na |  |  | 17544 | -1.275 | -0.3469 | Yes |
| 136 | SOCS1 | na |  |  | 17552 | -1.279 | -0.3389 | Yes |
| 137 | TNFSF10 | na |  |  | 17691 | -1.328 | -0.3374 | Yes |
| 138 | CASP4 | na |  |  | 17716 | -1.342 | -0.3299 | Yes |
| 139 | BTG1 | na |  |  | 17748 | -1.358 | -0.3227 | Yes |
| 140 | PSME2 | na |  |  | 17817 | -1.395 | -0.3171 | Yes |
| 141 | ISG20 | na |  |  | 17840 | -1.410 | -0.3090 | Yes |
| 142 | ST3GAL5 | na |  |  | 17841 | -1.411 | -0.2998 | Yes |
| 143 | BATF2 | na |  |  | 17919 | -1.450 | -0.2943 | Yes |
| 144 | ISOC1 | na |  |  | 17943 | -1.462 | -0.2860 | Yes |
| 145 | CD74 | na |  |  | 17961 | -1.469 | -0.2772 | Yes |
| 146 | IRF2 | na |  |  | 17974 | -1.475 | -0.2682 | Yes |
| 147 | PTGS2 | na |  |  | 17994 | -1.483 | -0.2595 | Yes |
| 148 | PSMB8 | na |  |  | 18223 | -1.614 | -0.2609 | Yes |
| 149 | IL6 | na |  |  | 18227 | -1.619 | -0.2505 | Yes |
| 150 | NMI | na |  |  | 18304 | -1.687 | -0.2434 | Yes |
| 151 | ARID5B | na |  |  | 18389 | -1.746 | -0.2364 | Yes |
| 152 | HLA-DRB1 | na |  |  | 18450 | -1.799 | -0.2278 | Yes |
| 153 | OAS2 | na |  |  | 18550 | -1.868 | -0.2208 | Yes |
| 154 | PSMB9 | na |  |  | 18892 | -2.341 | -0.2233 | Yes |
| 155 | IFI27 | na |  |  | 18923 | -2.408 | -0.2092 | Yes |
| 156 | BST2 | na |  |  | 18934 | -2.418 | -0.1938 | Yes |
| 157 | TXNIP | na |  |  | 18972 | -2.510 | -0.1794 | Yes |
| 158 | NAMPT | na |  |  | 18985 | -2.547 | -0.1633 | Yes |
| 159 | SOD2 | na |  |  | 18995 | -2.569 | -0.1470 | Yes |
| 160 | ST8SIA4 | na |  |  | 19018 | -2.638 | -0.1308 | Yes |
| 161 | GBP4 | na |  |  | 19050 | -2.740 | -0.1145 | Yes |
| 162 | CXCL11 | na |  |  | 19056 | -2.745 | -0.0968 | Yes |
| 163 | PSME1 | na |  |  | 19078 | -2.824 | -0.0794 | Yes |
| 164 | BPGM | na |  |  | 19126 | -3.042 | -0.0620 | Yes |
| 165 | B2M | na |  |  | 19139 | -3.164 | -0.0419 | Yes |
| 166 | C1S | na |  |  | 19156 | -3.351 | -0.0208 | Yes |
| 167 | VAMP8 | na |  |  | 19170 | -3.509 | 0.0015 | Yes |
Table: GSEA details [plain text format]

  

Fig 2: HALLMARK\_INTERFERON\_GAMMA\_RESPONSE      
 Blue-Pink O' Gram in the Space of the Analyzed GeneSet

  

Fig 3: HALLMARK\_INTERFERON\_GAMMA\_RESPONSE: Random ES distribution      
 Gene set null distribution of ES for **HALLMARK\_INTERFERON\_GAMMA\_RESPONSE**
